# Resisted Sprint Training Improves Overground Sprint, Jump, and Isometric Mid-Thigh Pull Kinetics and Kinematics in Male Youth Ice Hockey Players: A Randomized Control Trial

**DOI:** 10.5114/jhk/200549

**Published:** 2025-05-29

**Authors:** Martin Dietze-Hermosa, Samuel Montalvo, Matthew P. Gonzalez, Anna Briggs, Sandor Dorgo

**Affiliations:** 1 Department of Human Performance and Recreation, Brigham Young University-Idaho, Rexburg, USA.; 2 Wu Tsai Human Performance Alliance, Stanford University, Palo Alto, USA.; 3 Stanford Sports Cardiology, Division of Cardiovascular Medicine, Stanford University School of Medicine, Palo Alto, USA.; 4 Department of Kinesiology, The University of Texas at San Antonio, San Antonio, USA.

**Keywords:** jump, speed, skating, biomechanics

## Abstract

This study investigated the effects of an on-ice resisted sprint training (RST) intervention, an overground RST intervention and a traditional training control condition on measures associated with ice skating completion time. The vertical jump, the broad jump, the isometric mid-thigh pull, and overground sprint completion times, along wth sprint kinetics and kinematics were obtained prior and at the conclusion of the 8-week training intervention. There was a 7% increase in jump height (p < 0.05), a 9% increase in the jump peak force (p < 0.05), a 10% increase in jump peak power and a 21% increase in broad jump distance (p < 0.001) across all groups. Only the overground RST group significantly improved by 12% (p = 0.007) in the isometric mid-thigh pull peak force. All groups decreased 9.14-m completion time (−3%), 36.58-m completion time (−4%), and flying 30-m top speed completion time (−9%) (p < 0.05). The on-ice RST group improved by 22% in theoretical maximal horizontal force, 24% in theoretical maximal horizontal power, and 7% in the maximal ratio of force (p < 0.05). The step rate decreased by −2%, and the trunk angle increased by 48% at the touchdown and 30% at the toe-off for the overground RST group (p < 0.05). RST and bodyweight training induced comparable changes across most overground athletic performance measures associated with ice skating. Coaches desiring to improve overground predictors of ice skating performance in ice hockey players may benefit from incorporating RST as a component of a well rounded strength and conditioning program.

## Introduction

Maximal ice skating acceleration and speed are two of the main contributors to successful ice hockey performance (Farlinger et al., 2007; Stastny et al., 2023). There are certain commonly incorporated overground (off-ice) measures of athletic performance associated with ice skating acceleration and top speed. Those measures of athletic performance displaying the strongest associations include vertical jump height, broad jump distance, overground sprint speed and acceleration phase completion time (Farlinger et al., 2007; Roczniok et al., 2024; Runner et al., 2016). For example, Runner et al. (2016) found that an increase in vertical jump height, a commonly used measure of lower body muscular power, contributed to decreased ice skating completion time (*b* = −0.029, t_(35)_ = −2.680, *p* < 0.011) in collegiate male ice hockey players. In elite youth players, ice skating acceleration performance was related to vertical jump height (*r* = −0.46) and broad jump distance (r = −0.31) (Roczniok et al., 2024). In another study on male competitive ice hockey players, authors reported that ice skating completion time was associated with broad jump distance (*r* = −0.74), and vertical jump height (*r* = −0.71) (Farlinger et al., 2007). Blanàr et al. (2019) reported associations between the Weave test completion time (ice skating in a crossover manner) with power during the squat jump at 70% of body weight (*r* = −0.383), vertical jump height (*r* = −0.363), left single-leg lateral jump distance (*r* = −0.581), and right single-leg lateral jump distance (*r* = −0.563) in youth male ice hockey players. Consequently, it seems that lower body power as measured by jumping is associated with improved ice skating acceleration and speed.

Multiple studies have reported strong associations between overground speed and acceleration with ice skating speed and acceleration (Krause et al., 2012). For instance, Krause et al. (2012) reported that overground 36-m sprint completion time was strongly associated with ice skating completion time (*r* = 0.81) in high school male ice hockey players. Authors suggested that every one-second improvement in overground 36-m sprint time corresponded to a 0.6-s improvement in on-ice 36-m sprint time. Although seemingly small, this potential improvement in ice skating completion time can result in ice hockey players getting to the hockey puck faster and consequently achieving greater offensive and/or defensive opportunities. It is suggested that the association displayed between these athletic measures and ice skating speed and acceleration completion time can be attributed to the muscular power necessary for rapid propulsion during maximal ice skating and the importance of muscular power during jumping and overground sprinting (Farlinger et al., 2007). Hence, these overground tests, used as indicators of ice hockey performance potential, are applied to categorize the players’ skill level (Lemoyne et al., 2022). Therefore, coaches seek to improve these measures with overground training. Strength and conditioning practitioners suggest that an overground training program that includes sprint training and jumping exercises may have a positive impact on ice hockey players’ ice skating speed and acceleration (Galati et al., 2023).

Ice hockey training programs typically include overground training to improve muscular power, and locomotion speed and acceleration (Neeld, 2018). Previous studies support the transfer of overground training to improved ice hockey performance (Dæhlin et al., 2017; Naimo et al., 2015). One study indicated a reduction in maximal 33-m ice skating completion time (−5.2%; *d* = −1.15; *p* = 0.02) following a 4-week high intensity training regimen (Naimo et al., 2015). An 8-week combined plyometric and strength program was effective at substantially reducing 10-m ice skating completion time (−2.8%; *p* = 0.03) in high level ice hockey players (Dæhlin et al., 2017).

A popular modality utilized by strength and conditioning coaches to increase overground sprint acceleration and speed is resisted sprint training (Alcaraz et al., 2018; Loturco et al., 2023). Prior literature indicates that resisted sprint training in the form of sled towing was effective in increasing sprint acceleration in athletes of different running-based sports (Cahill et al., 2020). Interestingly, pertinent to ice hockey players, Thompson et al. (2020) revealed that a 15-kg overground resisted sprint was the primary predictor of ice skating sprint time (*R*^2^ = 0.62; *p* ≤ 0.001). Yet, there appears to be a paucity in the literature exploring the impact of a longitudinal resisted sprint training program in ice hockey players. A recent article reported positive effects of both overground and on-ice resisted sprint training to important ice skating performance measures (Dietze-Hermosa et al., 2024). Authors report that both resisted sprint training groups displayed improvements across all ice skating completion times (30-m top speed, s-cornering agility drill, 9.14-m, and 36.58-m acceleration ice skating sprint) with superior outcomes on certain ice skating tests compared to the control group. However, it is still unknown how an on-ice or an overground resisted sprint training program could impact important, frequently assessed, overground measures of ice hockey performance such as the vertical jump, the broad jump, and overground sprinting.

Therefore, the purpose of the study was to investigate the effects of an on-ice resisted sprint training intervention, an overground resisted sprint training intervention and a traditional training control condition on measures associated with ice skating completion time. It was hypothesized that both the on-ice and overground resisted sprint training programs would be effective at improving vertical jump height, broad jump distance, and overground sprint completion time. Additionally, it was expected that both resisted sprint training programs would display superior performance alterations in the vertical jump, the broad jump, and overground sprint completion times compared to the control.

## Methods

### Design and Procedures

In this randomized control trial, participants recruited from a local high school ice hockey team were randomly assigned to one of the three groups 1) eight weeks of on-ice resisted sprint training, 2) eight weeks of overground resisted sprint training, or 3) eight weeks of bodyweight training (control group). Participants completed a familiarization session during which all testing and training procedures were performed. During the familiarization session, the individualized sled load for the intervention was also determined. Then, during a separate session, participants completed pre-testing measures in randomized order consisting of vertical jumps, broad jumps, isometric mid-thigh pulls, and overground acceleration sprints. Following pre-testing, the randomized groups participated in their assigned training program. After the 8-week training programs, participants completed post-testing following the same design as outlined for pre-testing.

### Participants

Athletes were recruited from a local high school ice hockey club. The team consisted of players aged 14–18 years old. The teams’ roster consisted of 30 players, and it was anticipated that all of the players would participate in the study. The study was approved by the Birgham Young University-Idaho Institutional Review Board (approval code: IRB#:S21-13; approval date: 6 July 2021). Additionally, prior to the commencement of data collection, parents gave consent, athletes gave assent, and appropriate forms were collected by the research team. Participants were informed of the benefits and risks of the study prior to signing an institutionally approved documentation.

Using G*Power software (version 3.1, Universität Kiel, Germany) a repeated measures ANOVA *a priori* power analysis was conducted using sprint acceleration phase completion time data from Alcaraz et al. (2018) (Cohen’s *d* = 0.61) and an alpha of 0.05, indicating that with a sample of 30 players (10 per group) a statistical power of 0.83 would be reached. Due to illness, injury or relocation, a total of 24 players completed the study (8 per group) reducing the statistical power to 0.72.

### Measures

The participants’ body mass and height were measured with participants removing their shoes and standing with their back straight and the head in a neutral position. Body height was measured to the nearest centimeter and mass to the nearest hundredth of a kilogram.

The mid-thigh position during the isometric mid-thigh pull (IMTP) for each participant was determined before testing (Comfort et al., 2019). Participants maximally pulled upwards for three seconds while on the force plates (1,000 Hz; PASPORT force plate, PS-2142, PASCO Scientific, Roseville, CA, USA), and data were summed between the two force plates for analysis. The data were analyzed according to previous literature (Comfort et al., 2019). The variables of interest were the peak force and the rate of force development (RFD). Participants performed three trials with two minutes separating each trial with the average used for subsequent data analysis.

Vertical jumps were performed with participants standing on the force plates with one foot on each force plate (1,000 Hz; PASPORT force plate, PS-2142, PASCO Scientific, Roseville, CA, USA) and left and right force plate data were summed for analysis (Montalvo et al., 2021). The average of the three trials was used for analysis for all measures of interest. Data were analyzed and variables of interest were obtained following prior literature (Montalvo et al., 2021). Variables of interest for the vertical jump were estimated jump height, the modified reactive strength index, relative peak force, and relative peak power. In total, participants performed three vertical jump trials separated by a two-minute rest interval.

For the broad jumps, the distance was the primary variable of interest. Participants commenced standing with both feet behind a baseline, then they executed a forward countermovement jump (Dietze-Hermosa et al., 2021). Jump distance was measured as the distance from the takeoff to the back of the athlete’s heel (Dietze-Hermosa et al., 2021). Participants were allowed three attempts separated by a two-minute rest interval.

Participants completed three maximal 36.58-m (40 yard) sprints separated by a two-minute rest interval. Participants started in an athletic stance position at the 0-m mark, with the remote starter placed at the heel of the participant. Completion time was calculated as the time when the participant moved their heel away from the remote starter until they crossed the timing gates at 36.58 m (Dietze-Hermosa et al., 2021). Timing gates (TC-timer, Brower timing system, Draper, UT, USA) were used to accurately capture completion time during sprints with time recorded to the nearest 0.01 s. Moreover, to capture split time, timing gates were also placed at the 9.14-m mark (10 yard).

Particiapnts completed three maximal top speed sprints separated by a two-minute rest interval. Participants started in an upright standing position 40 m away from the first marker (timing gate; TC-timer, Brower timing system, Draper, UT, USA). After slowly gathering speed, athletes were at top speed once reaching the first marker and attempted to maintain top speed until the end marker set at a 30-m distance from the first marker (Dietze-Hermosa et al., 2021).

The participant’s sprint profile (F0, V0, P_max_, RF_max_, DRF and SFV) was obtained during 30-m acceleration sprints which enabled creation of the force-velocity curve and accurate estimation of certain kinetics during sprinting (Dietze-Hermosa et al., 2021; Dylan et al., 2019). The details of the methodology can be found elsewhere (Morin et al., 2019; Samozino et al., 2016). Sprint profile components can be obtained and calculated using a single high-speed camera system (240 fps) and markers placed at specific distances (5, 10, 15, 20, 25, and 30 m) (Romero-Franco et al., 2017). An iPad Air (Apple Inc., Cupertino, CA, USA, 240 fps) was used for data collection.

A single high-speed camera with a capturing frequency of 240 Hz (same utilized to capture the Sprint Profile) was placed on a stationary stand with the optical axis perpendicular to the movement plane of the lower limbs during sprinting enabling the accurate identification of joint angular kinematic variables and spatiotemporal variables of interest (step length, step rate, flight time, contact time). The captured video file was imported into Kinovea software (v.0.9.5) for kinematic analysis. The methodology proposed by prior literature for joint angle calculations and spatiotemporal variables of interest (flight time, contact time, step length, step frequency) was followed (Damsted et al., 2015; Lahti et al., 2019; Zabaloy et al., 2020).

Athletes were randomly assigned to one of the three groups (an on-ice intervention, an overground training intervention, or to a pseudo-control group). The overground RST and pseudo-control groups completed training interventions in a local school gym with finished hardwood flooring. The overground RST group completed two sessions a week of 6–9 maximal sprints pulling a loaded sled over a 20-m distance, with participants given three minutes of rest between each resisted sprint. The pseudo-control group engaged in the prescribed training program of the sport coaching staff, since the omission of any training session would likely induce detraining effects and result in performance decrements. For the sake of brevity and simplicity, this group will be referred to simply as the “control group” throughout the article. The on-ice RST group also participated in two weekly sessions of 6–9 maximal skating sprints over a 20-m distance. All training groups engaged in their designated program for the duration of 8 weeks. The training protocol, including specific training program details (sled-load, distance, frequency, volume, and duration), followed that outlined in Dietze-Hermosa et al. (2024).

### Statistical Analyses

All data collected for variables of interest were restructured in a comprehensive Microsoft Excel spreadsheet and imported into JASP software (JASP Team [2023]. JASP (Version 0.17.3) [Computer software]) based in R statistical programming language for statistical processing. Assumptions of normality were verified using the Shapiro-Wilk tests and visual assessment of distribution through a histogram. The test-retest reliability of each variable of interest was determined using the intraclass correlation coefficient (ICC, two-way mixed effects, absolute agreement) with 95% confidence intervals. ICCs were interpreted as: <0.40 = poor; 0.40–0.60 = fair; 0.60–0.75 = good; 0.75–1.00 = excellent, like in prior sprint literature (Koo and Li, 2016).

Variables of interest displayed acceptable test-retest reliability and data distribution; thus, the average value was used for statistical analysis. The Levene's test was used to ensure the data met the criteria for homogeneity of variance. To establish the effects of the intervention on outcome variables, a series of two-way mixed factorial ANOVAs (3 groups x 2 time-points) were performed. Significant interactions were decomposed using Holm-Bonferroni adjusted pairwise comparisons. Moreover, unstandardized effect sizes from the repeated measures ANOVA were computed as partial eta squared (η_p_^2^) and interpreted as: small (η_p_^2^ = 0.01); medium (η_p_^2^ = 0.09); and large (η_p_^2^ = 0.25) effects (Lakens, 2013). Additionally, Cohen’s *d* was calculated. The effect sizes were interpreted as: trivial = ≤ 0.20; small = 0.20 to 0.60; moderate = 0.60 to 1.2; large = 1.2 to 2.0; very large = 2.0 to 4.0; nearly perfect >4.0 (Hopkins et al., 2009). All data were analyzed at a significance level of 0.05.

## Results

**Figure 1:**
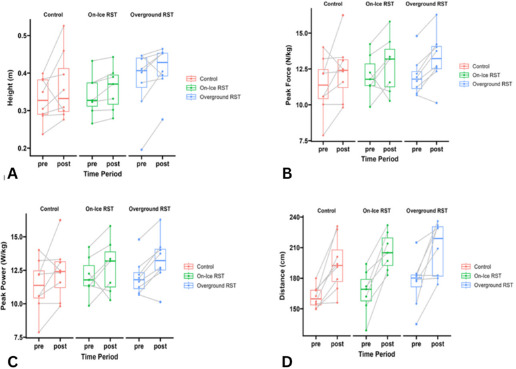
A = changes in countermovement jump height across groups; B = changes in countermovement jump peak force across groups; C = changes in countermovement jump peak power across groups; D = changes in broad jump distance across groups.

There was a significant main effect for the time point on CM jump height [F(1,21) = 7.192; *p* = 0.014; η_p_^2^ = 0.264; large] (Figure 1A). This corresponded to a 7% increase in jump height across all groups (Cohen’s *d* = 0.56; *p* = 0.014; small). There was also a main effect of the time point on peak force [F(1,21) = 12.098; *p* = 0.002; η_p_^2^ = 0.377; large], and peak power [F(1,21) = 40.802; *p* < 0.001; η_p_^2^ = 0.671; large] (Figure 1B–1C). Consequently, there was a 9% increase in peak force (Cohen’s *d* = 0.73; *p* = 0.002; moderate) and a 10% increase in peak power (Cohen’s *d* = 1.33; *p* < 0.001; large) across all groups. There was a significant main effect for the time point on jump distance [F(1,21) = 58.95; *p* < 0.001; η_p_^2^ = 0.747; large] (Figure 1D). This corresponded to a total improvement of a 21% (35 cm) increase in broad jump distance across all groups (Cohen’s *d* = 1.60; *p* < 0.001; large).

There was a significant group*time point interaction effect for peak force [F(2,21) = 5.651; *p* < 0.011; η_p_^2^ = 0.361; large]. Post-hoc analyses indicated pre- to post-testing changes for the overground RST group (mean difference = 183.81 N; *p* = 0.007; Cohen’s *d* = 0.50; small) and for the on-ice RST group (mean difference = 150.69 N; *p* = 0.065; Cohen’s *d* = 0.40; small).

**Figure 2:**
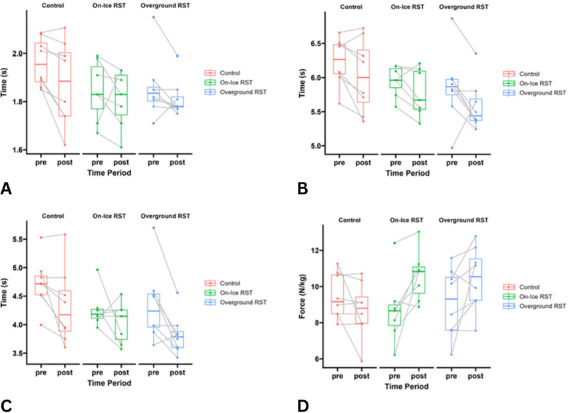
A = changes in 9.14-m sprint completion time across groups; B = changes in 36.58-m sprint completion time across groups; C = changes in 30-m flying sprint completion time across groups; D = changes in theoretical maximal horizontal force across groups.

There was a significant main effect of the time point on 9.14-m completion time [F(1,21) = 7.445; p = 0.013; η_p_^2^ = 0.271; large; 0.06 s], 36.58-m completion time [F(1,21) = 10.406; p = 0.004; η_p_^2^ = 0.342; large; 0.222 s], and 30-m top speed completion time [F(1,21) = 15.256; p < 0.001; η_p_^2^ = 0.433; large; 0.387 s] (Figure 2A–2C).

**Table 1:** Athlete’s descriptive information by group and time.

	PRE Testing						POST Testing						Pairwise Comparison		
	Control		Overground		On-Ice		Control		Overground		On-Ice		Control	Overground RST	On-Ice RST
	Mean	SD	Mean	SD	Mean	SD	Mean	SD	Mean	SD	Mean	SD	Mean Difference (95% CI)	Mean Difference (95% CI)	Mean Difference (95% CI)
Body height (m)	1.72	0.10	1.78	0.04	1.71	0.11	1.74	0.12	1.78	0.04	1.72	0.13	−0.02 (−0.08, 0.00)	0.00 (−0.09, 0.00)	−0.01 (−0.10, 0.00)
Body mass (kg)	65.66	10.89	69.48	8.01	59.67	17.90	66.19	11.21	69.98	8.35	60.43	17.39	−0.53 (−1.11, 0.00)	−0.50 (−1.26, 0.00)	−0.86 (−1.45, 0.00)
BMI (kg/m^2^)	22.06	2.59	21.85	2.23	20.04	3.75	22.10	2.42	22.06	2.21	20.74	3.79	−0.04 (−0.09, 0.00)	−0.21 (−0.42, 0.00)	−0.70 (−1.32, 0.00)
Sled load (kg)	-	-	34.10	4.54	43.10	6.80		-	-	-	-	-	-	-	-

Note: m = meters; kg = kilograms; BMI = body mass index; CI = confidence interval

**Table 2:** Vertical and broad jump descriptive information by group and time.

	PRE Testing						POST Testing						Pairwise Comparison		
	Control		Overground		On-Ice		Control		Overground		On-Ice		Control	Overground	On-Ice
	Mean	SD	Mean	SD	Mean	SD	Mean	SD	Mean	SD	Mean	SD	Mean Difference (95% CI)	Mean Difference (95% CI)	Mean Difference (95% CI)
Broad Jump Distance (cm)	161	10	176	23	166	22	194	26	209	26	206	18	32 (7, 58)	33 (7, 58)	40 (12, 67)
CMJ Jump Height (m)	0.33	0.06	0.38	0.09	0.34	0.06	0.36	0.09	0.41	0.06	0.36	0.06	0.04 (−0.01, 0.09)	0.03 (−0.03, 0.09)	0.02 (−0.04, 0.08)
CMJ Peak Force (N/kg)	11.33	1.97	12.00	1.33	12.04	1.45	12.36	2.03	13.25	1.78	12.79	1.98	1.03 (−0.61, 2.67)	1.25 (−0.39, 2.89)	0.75 (−0.98, 2.50)
CMJ Peak Power (W/kg)	47.75	6.73	51.96	9.01	47.95	6.61	52.98	8.55	57.17	7.00	51.67	6.57	5.24 (1.07, 9.40) **	5.21 (1.05, 9.37) **	3.71 (−0.74, 8.16)
CMJ RSImod	0.32	0.14	0.30	0.10	0.32	0.06	0.28	0.09	0.30	0.11	0.30	0.08	−0.04 (−0.20, 0.12)	−0.01 (−0.17, 0.16)	−0.02 (−0.19, 0.15)

Note: ** = Significant at p = 0.01; CMJ = countermovement jump; RSImod = reactive strength index modified; cm = centimeters; m = meters; N = newtons; W = Watts; s = seconds; kg = kilograms; CI = confidence interval

**Table 3:** Overground sprint completion time, kinematic, and kinetic descriptive information by group and time.

	PRE Testing						POST Testing						Pairwise Comparison		
	Control		Overground		On-Ice		Control		Overground		On-Ice		Control	Overground	On-Ice
	Mean	SD	Mean	SD	Mean	SD	Mean	SD	Mean	SD	Mean	SD	Mean Difference (95% CI)	Mean Difference (95% CI)	Mean Difference (95% CI)
Overground Completion Time (9.14-m) (s)	1.96	0.10	1.86	0.13	1.85	0.12	1.88	0.17	1.81	0.08	1.81	0.12	−0.09 (−0.20, 0.02)	−0.04 (−0.16, 0.07)	−0.03 (−0.16, 0.09)
Overground Completion Time (36.58-m) (s)	6.23	0.35	5.86	0.52	5.95	0.23	6.02	0.53	5.58	0.36	5.78	0.35	−0.21 (−0.60, 0.18)	−0.28 (−0.67, 0.11)	−0.17 (−0.59, 0.24)
Overground Completion Time (30-m Top Speed) (s)	4.72	0.43	4.35	0.65	4.27	0.33	4.32	0.66	3.82	0.35	4.04	0.36	−0.40 (−0.96, 0.16)	−0.53 (−1.09, 0.03)	−0.23 (−0.83, 0.37)
Overground Contact Time (s)	0.10	0.01	0.10	0.01	0.10	0.01	0.09	0.02	0.10	0.01	0.09	0.01	−0.01 (−0.02, 0.01)	0.00 (−0.01, 0.01)	−0.01 (−0.02, 0.00)
Overground Flight Time (s)	0.15	0.01	0.14	0.01	0.15	0.02	0.15	0.02	0.14	0.01	0.15	0.02	0.00 (−0.02, 0.02)	0.01 (−0.01, 0.03)	0.00 (−0.02, 0.02)
Overground 30-m Sprint Theoretical Horizontal Force (N/kg)	9.48	1.24	9.10	1.90	8.71	1.91	8.69	1.53	10.42	1.74	10.59	1.40	−0.79 (−2.69, 1.11)	1.32 (−0.58, 3.22)	1.89 (−0.15, 3.92) *
Overground 30-m Sprint Theoretical Horizontal Velocity (m/s)	7.01	0.46	7.48	0.59	7.15	0.45	7.34	0.56	7.57	0.55	7.26	0.54	0.34 (−0.13, 0.80)	0.09 (−0.38, 0.55)	0.11 (−0.39, 0.61)
Overground 30-m Sprint Theoretical Horizontal Power (W/kg)	16.70	2.87	17.21	3.43	15.42	3.29	16.09	3.70	19.50	2.99	19.09	2.75	−0.61 (−3.72, 2.50)	2.29 (−0.83, 5.40)	3.68 (0.35, 7.01) *
Overground 30-m Sprint Force-Velocity Slope	−1.35	0.15	−1.22	0.26	−1.20	0.31	−1.18	0.16	−1.38	0.28	−1.45	0.19	0.17 (−0.15, 0.50)	−0.16 (−0.49, 0.16)	−0.25 (−0.60, 0.10)
Overground 30-m Sprint Maximal Ratio of Force	0.43	0.03	0.44	0.03	0.42	0.03	0.43	0.04	0.46	0.03	0.45	0.02	−0.00 (−0.03, 0.02)	0.02 (−0.01, 0.04)	0.04 (0.01, 0.06) **
Overground 30-m Sprint Decrease in Maximal Ratio of Force	−0.13	0.02	−0.12	0.03	−0.12	0.03	−0.11	0.02	−0.13	0.03	−0.14	0.02	0.02 (−0.02, 0.05)	−0.01 (−0.04, 0.02)	−0.02 (−0.05, 0.02)

Note: * = significant at p = 0.05; ** = significant at p = 0.01; m = meters; N = newtons; W = Watts; s = seconds; CI = confidence interval

**Table 4:** Overground sprint kinematic descriptive information by group and time.

	PRE Testing						POST Testing						Pairwise Comparison		
	Control		Overground		On-Ice		Control		Overground		On-Ice		Control	Overground	On-Ice
	Mean	SD	Mean	SD	Mean	SD	Mean	SD	Mean	SD	Mean	SD	Mean Difference (95% CI)	Mean Difference (95% CI)	Mean Difference (95% CI)
Overground Sprint Trunk Angle at Take-Off (degrees)	15.17	6.39	19.13	5.57	18.86	3.73	15.30	5.43	24.85	9.33	18.34	3.25	0.13 (−7.34, 7.60)	5.73 (−1.75, 13.20)	−0.51 (−8.50, 7.47)
Overground Sprint Hip Swing Angle at Take-Off (degrees)	78.23	11.18	85.97	8.44	82.14	8.85	76.26	10.72	82.89	8.15	80.66	5.50	−1.97 (−11.49, 7.56)	−3.08 (−12.60, 6.44)	−1.49 (−11.67, 8.69)
Overground Sprint Knee Swing Angle at Take-Off (degrees)	76.77	13.20	71.28	11.73	75.04	15.55	81.47	12.62	78.41	5.30	76.93	16.13	4.70 (−4.32, 13.71)	7.13 (−1.89, 16.14)	1.89 (−7.75, 11.52)
Overground Sprint Hip Stance Angle at Take-Off (degrees)	18.34	7.48	15.13	7.50	14.71	7.05	21.00	5.97	11.64	11.24	16.37	4.65	2.67 (−8.45, 13.78)	−3.49 (−14.60, 7.63)	1.66 (−10.22, 13.54)
Overground Sprint Knee Stance Angle at Take-Off (degrees)	162.45	6.50	162.42	4.31	159.96	8.89	163.85	4.34	165.53	5.82	158.56	6.99	1.40 (−4.61, 7.41)	3.11 (−2.90, 9.13)	−1.39 (−7.82, 5.04)
Overground Sprint Trunk Angle at Touchdown (degrees)	16.85	5.39	17.96	7.18	19.31	1.41	17.16	5.73	26.66	8.95	20.03	2.99	0.31 (−7.14, 7.77)	8.71 (1.25, 16.16) *	0.71 (−7.26, 8.69)
Overground Sprint Hip Swing Angle at Touchdown (degrees)	55.33	5.57	59.09	11.25	59.16	5.30	52.43	8.74	61.41	10.88	55.37	9.26	−2.90 (−9.68, 3.89)	2.33 (−4.46, 9.11)	−3.79 (−11.04, 3.46)
Overground Sprint Knee Swing Angle at Touchdown (degrees)	142.16	5.77	140.44	6.29	143.06	4.06	146.54	5.61	146.36	7.35	146.68	8.94	−4.38 (−12.32, 3.57)	−5.91 (−13.86, 2.03)	−3.61 (−12.11, 4.88)
Overground Sprint Hip Stance Angle at Touchdown (degrees)	2.24	6.69	3.48	8.26	3.83	9.34	−2.68	11.81	−9.64	12.16	−7.14	8.36	−4.92 (–18.82, 8.98)	−13.12 (−27.01, 0.77)	−10.97 (−25.83, 3.89)
Overground Sprint Knee Stance Angle at Touchdown (degrees)	84.04	6.53	84.65	7.44	92.25	14.83	81.65	11.89	79.53	10.42	84.23	14.88	−2.39 (−11.99, 7.22)	−5.13 (14.73, 4.48)	−8.02 (−18.29, 2.25)
Overground Step Length (cm)	134.06	9.33	143.28	12.24	130.56	10.11	135.68	9.07	146.04	10.00	132.52	10.24	1.62 (−4.66, 7.90)	2.76 (−3.52, 9.04)	1.96 (−4.75, 8.68)
Overground Step Rate (steps/s)	4.10	0.42	4.24	0.11	4.10	0.26	4.14	0.39	4.15	0.17	4.23	0.30	0.04 (−0.11, 0.20)	−0.09 (−0.24, 0.06) *	0.12 (−0.4, 0.28)

Note: * = significant at p = 0.05; ** = significant at p = 0.01; cm = centimeters; s = seconds; CI = confidence interval

There was a significant group*time point interaction effect on the step rate [F(2,21) = 5.014; *p*=0.017; η_p_^2^ = 0.334; large]. Follow-up analysis revealed a moderate decrease for the overground RST group (Cohen’s *d* = 0.64; moderate). There was significant group*time point interaction effect on the trunk angle at the touchdown [F(2,21) = 4.387; *p* = 0.026; η_p_^2^ = 0.305; large]. Follow-up analyses revealed an increased trunk angle for the overground RST group (mean difference = 8.706; *p* = 0.014; Cohen’s *d* = 1.07; moderate). There was a significant main effect for the time point on contact time [F(1,21) = 8.310; *p* = 0.009; η_p_^2^ = 0.310; large; 0.005 s]. This corresponded to a moderate effect size (Cohen’s *d* = 0.62; *p* < 0.007).

There were significant group*time point interaction effects on theoretical maximal horizontal force [F(2,21) = 5.857; *p* = 0.010; η_p_^2^ = 0.369; large] (Figure 2D), theoretical maximal horizontal power [F(2,21) = 5.211; *p* = 0.015; η_p_^2^ = 0.343; large], the force-velocity slope [F(2,21) = 4.948; *p* = 0.018; η_p_^2^ = 0.331; large], and the maximal ratio of force [F(2,21) = 5.938; *p* = 0.013; η_p_^2^ = 0.351; large]. Follow-up analyses indicated significant pre- to post-testing changes for the on-ice RST group on theoretical maximal horizontal force (mean difference = 1.886 N/kg; *p* = 0.046; Cohen’s *d* = 1.13; moderate), theoretical maximal horizontal power (mean difference = 3.679 W/kg; *p* = 0.016; Cohen’s *d* = 1.21; large), and the maximal ratio of force (mean difference = 0.038; *p* = 0.004; Cohen’s *d* = 1.32; large). There was a significant main effect for the time point on theoretical maximal velocity [F(1,21)= 4.668; *p* = 0.043; η_p_^2^ = 0.189; moderate; 0.178 m/s]. Across all groups this equated to a small effect size (Cohen’s *d* = 0.34; *p* = 0.04).

## Discussion

This study aimed to compare the effects of an 8-week on-ice resisted sprint training program, an 8-week overground resisted sprint training program, and a control condition on overground athletic measures associated with ice skating in ice hockey players. Vertical jump height (7%), vertical jump peak force (9%), vertical jump peak power (10%), and broad jump distance (21%) increased across all three training groups. Moreover, 9.14-m (3%) and 36.58-m (4%) overground acceleration sprint completion times as well as 30-m top speed (9%) completion time improved across all groups. Thus, the study hypothesis was partially supported given the increase in jump measures with simultaneous reduction in overground sprint completion times across all groups; yet superior findings for the resisted sprint training groups were not observed.

Study findings agree with prior literature demonstrating increases in vertical jump measures after participating in overground RST (Gil et al., 2018). After 6 weeks of overground RST, soccer players demonstrated increased vertical jump height (15%) (Gil et al., 2018). Gil and colleagues (2018) also reported that the control group who participated in unresisted sprint training exhibited similar improvements (15%) to the overground RST intervention group. In youth tennis players, after engaging in 6 weeks of overground RST, players reported increases in vertical jump height (5%) and broad jump distance (5%), yet this was not different from the body weight training group (vertical jump height = 6%; broad jump distance = 3%) (Moya-Ramon et al., 2020). The present study findings indicate no significant differences in vertical jump height, peak force, and peak power improvement between the overground RST and control groups. The lack of differences between the overground RST and control groups in the current study was not unexpected as previous research had indicated improved jumping following plyometric, strength, bodyweight, and non-resisted sprint training (Petrakos et al., 2016).

We observed a 21% increase in broad jump distance across all groups. Prior literature supports improvements in broad jump performance after overground RST (Cahill et al., 2020; Moya-Ramon et al., 2020). The greater improvement in broad jump distance compared to vertical jump height, as a relative amount, may be a consequence of the horizontal direction of force application during RST compared to vertical jumping (Cahill et al., 2020; Dylan et al., 2019). To our knowledge, this is the first study to demonstrate that jumping measures (both vertical and horizontal) can be improved by engaging in on-ice RST. Prior research suggests that RST can target the mechanisms that underpin the stretch-shortening cycle, thus resulting in improvements during plyometric exercise, such as vertical and broad jumping (Harrison and Bourke, 2009).

Various studies report meaningful improvements in overground sprint completion times by a tenth of second or more following RST (Alcaraz et al., 2018). In the present study, all groups improved in overground 9.14-m completion time (0.06 s; 3%), 36.58-m completion time (0.222 s; 4%), and 30-m top speed completion time (0.387 s; 9%). Cahill and colleagues (2020) reported decreases in overground 10-m (Cohen’s *d* = 1.05; moderate) and 20-m (Cohen’s *d* = 1.03; moderate) completion times after an 8-week overground RST program in high school athletesFormatting.... Our study findings are further supported by authors who reported a 3% reduction in overground 10-m completion time and a 2% reduction in 30-m completion time after engaging in an 8-week overground RST program with 40% body weight (Rodríguez-Rosell et al., 2020). However, there exist conflicting results regarding the magnitude of sprint completion time improvement when RST is compared to control or unresisted sprint training (Alcaraz et al., 2018). Authors noting lack of differences between groups suggest that the magnitude of training loads may have been insufficient to induce changes above those observed by the unresisted sprinting or control training groups (Gil et al., 2018). Another potential explanation for lack of differences between groups may be that training one component of muscle capacity by the control group (power with high velocity body weight exercises; i.e., vertical jumps, push-ups, lunges) may translate over to increased overground sprint completion times (Prieske et al., 2018). As will be discussed in subsequent paragraphs, when considering RST specifically, in addition to increasing ground forces applied horizontally, RST is proposed to increase leg muscle strength during the stance phase potentially leading to decreased contact time and an increased step rate. The present study observed a decrease in contact time across all groups, with increased horizontal force and power for RST groups. These changes are proposed to drive the reduced sprint completion time noted after RST and may partially explain the RST findings of the present study (Alcaraz et al., 2018; Petrakos et al., 2016).

High levels of peak isometric strength may be an important quality for ice hockey players to possess. Small peak force increases during the IMTP were observed for the overground RST group (mean difference = 183.81 N; *p* = 0.007; 12.20%) and the on-ice RST group (mean difference = 150.69 N; *p* = 0.065; 12.75%). To the authors knowledge, this is the first study to explore the impact of resisted sprint training on IMTP measures in youth athletes. Despite challenges to contrast findings to other literature, studies report a strong association between peak force during the IMTP and sprint completion times (r = −0.53 to −0.69) (Brady et al., 2019). Given this relationship, it is logical that increases in the IMTP peak force could be accompanied by improvements in overground sprint completion times induced by resisted sprint training.

We observed that both RST groups improved in overground sprint theoretical maximal horizontal force (overground RST: Cohen’s *d* = 0.72; 15%; on-ice RST: Cohen’s *d* = 1.13; 22%), theoretical maximal horizontal power (overground RST: Cohen’s *d* = 0.71; 13%; on-ice RST: Cohen’s *d* = 1.21; 24%), and the maximal ratio of force (overground RST: Cohen’s *d* = 0.57; 3%; on-ice RST: Cohen’s *d* = 1.32; 9%). After 8 weeks of overground RST, male youth athletes exhibited improvements in maximal horizontal force (Cohen’s *d* = 0.51; small) and maximal horizontal power (Cohen’s *d* = 0.51; small), corroborating the present study findings (Cahill et al., 2020; Edwards et al., 2022). Recently, Edwards and colleagues (2022) reported improvements in maximal horizontal force (Hedge’s *g* = 0.63; moderate), horizontal power (Hedge’s *g* = 1.04; moderate), and the maximal ratio of force (Hedge’s *g* = 0.99; moderate) following a 10-week heavy RST program in junior rugby players (Edwards et al., 2022). RST is known to target horizontal force application, thus improved overground horizontal force and power were anticipated (Dylan et al., 2019). The increased maximal ratio of force suggests that athletes applied force in a more horizontal oriented direction compared to pre-testing, which was considered more efficient during the initial acceleration steps of sprinting (Dylan et al., 2019). The first few steps of overground sprinting are high-force dependent. Alterations in maximal horizontal force, maximal horizontal power and the maximal ratio of force could underpin improvements seen in overground completion times (Cahill et al., 2020; Rabita et al., 2015). Development of these components are vital since they are considered the premier components determining overground sprint and ice skating speeds (Perez et al., 2020; Rabita et al., 2015). For instance, Perez and colleagues (2020) observed a very large correlation between maximal horizontal power and ice skating 40-m split time (r = −0.91; *p* < 0.001). Interestingly, the on-ice RST improvements appeared to translate into kinetic changes observed during overground sprinting.

Others have demonstrated overground training can translate into ice skating improvements (Dietze-Hermosa et al., 2024; Novák et al., 2019). This is the first study to suggest the carry-over of on-ice training to overground sprint kinetics.

Overground step contact time decreased across all groups (0.005 s; Cohen’s *d* = 0.62; 5%) albeit with the on-ice RST group appearing to have the greatest reduction (0.009 s). Prior research indicates reductions in contact time of up to 0.020 s following RST (Petrakos et al., 2016). Intuitively, a reduction in contact time typically results in a decreased sprint completion time via faster acceleration made by the athlete. Others indicate non-significant differences in contact time following overground RST (Lahti et al., 2019). The contrasting findings in these studies may be attributed to the loading scheme utilized across studies (Alcaraz et al., 2018).

In our study, there was an increase in the trunk angle at the touchdown for the overground RST group (mean difference = 8.706; Cohen’s *d* = 1.07; 48%). Other studies also report increased trunk angles (16–58%) after engaging in overground RST (Alcaraz et al., 2018; Petrakos et al., 2016). An increased trunk angle is purported to aid athletes apply forces in a more horizontal direction thereby improving sprint completion times. Observed trunk angle changes agree with the sprint kinetic modifications for RST groups in the present study (i.e., increased maximal horizontal force, maximal horizontal power, and the maximal ratio of force).

The study's limitation lies in the participants’ limited strength and conditioning experience. This limitation may have hindered the study's ability to fully reveal the effects of various training interventions on the outcome measures. The findings are specific to male youth ice hockey players, thus caution should be exercised when generalizing to other populations.

## Conclusions

In the present study, all training groups improved certain measures associated with ice skating completion time (i.e., vertical jump height, broad jump distance, overground sprint completion times). However, only the RST groups improved in 1) overground sprint (kinetic) profile components, 2) altered certain kinematics during sprinting, and 3) increased peak force during the IMTP. Therefore, our findings suggest that RST and bodyweight training induce comparable changes across most athletic performance measures associated with ice skating. Coaches desiring to improve off-ice predictors of ice skating performance (i.e., vertical jump height, broad jump distance, and overground sprint completion times) in ice hockey players may benefit from incorporating RST as a component of a well-rounded strength and conditioning program.
